# Identification of rehabilitation needs after a stroke: an exploratory study

**DOI:** 10.1186/1477-7525-2-53

**Published:** 2004-09-21

**Authors:** Lise R Talbot, Chantal Viscogliosi, Johanne Desrosiers, Claude Vincent, Jacqueline Rousseau, Line Robichaud

**Affiliations:** 1 Nursing Department, Faculty of Medicine, Université de Sherbrooke, 3001, 12^e ^Avenue Nord, J1H 5N4 Sherbrooke (Québec), Canada; 2 Research Centre on Aging, Faculty of Medicine, Université de Sherbrooke, Canada; 3 Occupational Therapy Department, Université Laval, Québec, G1K 7P4 Canada; 4 Occupational Therapy Department, Faculty of Medicine, Université de Montréal, H3C 3J7 Canada

**Keywords:** stroke, needs, rehabilitation, community reintegration, impairment, disability

## Abstract

**Background:**

Services to meet adequate rehabilitation needs of elderly stroke survivors are not always provided. Indeed, since 1995, in the wake of the Quebec shift to ambulatory care, home care services, mainly those related to rehabilitation of the elderly, are either unavailable or incomplete. The aim of this study was to examine the rehabilitation needs of this clientele from their hospitalization to their reintegration into the community.

**Methods:**

The "Handicap Production Process" conceptual approach was chosen to help identify the rehabilitation needs of persons affected by physical or cognitive disabilities due to the interactions between personal and environmental factors, and (activities of daily living, social roles). This qualitative exploratory study was performed in 2003. Data were collected among four groups of experts: patients, caregivers, health care providers and administrators. Data triangulation was used to ensure a rigorous analysis and validity of the results.

**Results:**

Unfulfilled needs could be found in the categories of pertaining to residence, community living, psychological and emotional needs. Indeed, it appears that a psychological follow-up to discuss acceptance and consequences of non-acceptance would facilitate mid-to long-term rehabilitation.

**Conclusion:**

Improving accessibility to healthcare services, respecting priority parking spaces for the disabled as well as promoting public awareness would enable a better social reintegration and recovery of social roles, thus limiting the onset of handicap situations.

## Background

After a stroke, a good proportion of the elderly rapidly re-enter the community without having benefited from rehabilitation services to help reduce their impairments and disabilities. Indeed, in Canada, only about 10% of stroke sufferers have access to intensive rehabilitation services [1]. The others, being relatively independent in their activities of daily living (walking, dressing, eating), return home with or without support services. However, aside from physical problems, there often are less noticeable disorders of a perceptive-cognitive nature (hemineglect, attention or organizational problems, and impaired learning ability). These disorders could trigger handicap situations [2] and be a source of subsequent losses of autonomy, which in turn can increase use of health services, recurring hospitalizations and premature institutionalization, resulting in an expensive health system. Moreover, a person's disabilities almost always affect the lives of his/her/family.

The World Health Organization [3] defines rehabilitation as the combination and coordination of medical, social, educational and vocational resources aimed at optimizing a person's functional independence. Rehabilitation methods are essentially intended to reduce a person's disabilities and prevent the onset of disabling situations in order to support an optimal quality of life. Local community service centers (CLSC) are deeply concerned that they are unable to assume their role of providing, for their elderly users, front-line services that should cover various areas such as prevention, screening, general services and social reintegration [4]. The shift to ambulatory care initiated in Quebec in 1995 did not result in the development of outpatient rehabilitation services, even though these services, regarded as essential, can noticeably reduce hospitalization and improve quality of life. The outpatient clientele is more often left to fend for itself and sometimes has to turn to private clinics. For those with insurance coverage, the costs of these services are partly reimbursed; but 1.5 million Quebecois (23%) do not own private health insurance. Moreover, there is no comparable information on the waiting delays of private clinics that would allow to evaluate accessibility to their rehabilitation services [5]. Because of that lack of information, the Ministry of Health and the Regional Health and Social Services Boards can hardly provide cost-effective human resources in rehabilitation and equitably distribute financial resources, all the while keeping in perspective intra-regional as well as inter-regional needs. In order to delimit these issues from different perspectives, this study examines the rehabilitation needs of stroke patients in relation to their being cared for in their own home and according to their capabilities and their. This research was based on the "Handicap Production Process" conceptual approach (HPP) [6,7].

### Handicap Production Process conceptual approach (HPP)

**Figure 1:**
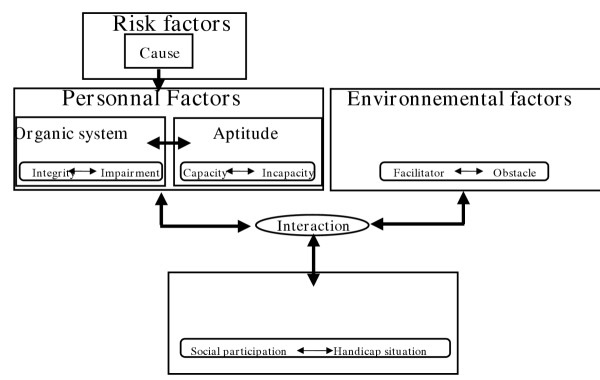
Handicap production process conceptual approach [8,9]

The HPP (handicap production process) ensues from the works of the International Network of the Handicap Production Process (Figure 1) – formerly known as the Quebec Committee for the International Classification of Impairment, Disability and Handicap (QC-ICIDH) [6,7] – which followed those of the World Health Organization [8]. This anthropologic model, used in research as well as in clinical situations, holds four components: risk factors (causes), personal factors (body systems and aptitudes), environmental factors (facilitator and obstacle) and (social participation and handicap situation).

Personal factors relate to the person's basic characteristics like age, sex, socio-cultural identity, body systems (integrity vs. impairment) and the aptitudes (capacities vs. disabilities). Body systems identify components of the whole body. Integrity of these systems is based on the human biological norms while an impairment relates to a degree of anatomical, or physiological damage. Aptitudes are associated with a person's capacity for physical or mental activity such as walking or understanding. The value of an aptitude is measured on a scale ranging from optimal capacity to total disability. In the HPP, ensure the survival and thriving of an individual within society, during his/her entire life. They are arranged in twelve categories, the first six referring to activities of daily living and the last six relating to social roles valued by the person or his/her socio-cultural context. These categories are: nutrition, body condition, personal care, speech, habitation, mobility, responsibilities, interpersonal relationships, community living, leisure activities as well as education and work. The environmental factors are the constituents of a person's surroundings than can affect the realization of a living habit. Interactions between personal and environmental factors create needs that impact a person in the execution of his/her activities of daily living, thus limiting his/her social participation. A positive interaction between personal and environmental factors supports social participation while a negative interaction produces the development of handicap situations.

### Definition of needs

Needs are subjective, since they are felt by the person (Talbot L. *Les besoins de santé des individus*. Unpublished manuscript). It could be a need for a resource to provide adequate and ample services. Bradshaw's taxonomy [9], used by Pineault and Daveluy [10], distinguishes four types of needs: normative, perceived, expressed and comparative. Normative needs are those that agree with norms, as defined by health professionals. Perceived needs are those perceived by individuals, depending on health services available. They become expressed needs, once articulated. Generalization of evaluated needs in a population results in comparative needs. Finally, needs depend on factors related to the person and his/her environment, on organizational factors, on factors related to the service providers [11]. As observed by repeated measures done in the post-stroke period, needs evolve with time, as do their response, depending on which services were provided. Furthermore, existing definition of needs seem to justify resource constraints rather than to satisfy health care needs of the person. The gap between perceived needs and normative needs is an area of improvement in the quality of services [21].

According to the HPP, rehabilitation needs would result from a discrepancy between the capacities of the person and the different factors of his/her environment [12]. These rehabilitation needs are associated with the handicap situations of the person in doing his/her daily activities, as compared to the most desirable level. Needs can therefore be multilevel (personal or environmental factors).

Using four groups of people (patients, caregivers, health care providers and administrators), the aim of this research was to investigate the expressed and normative rehabilitation needs of post-stroke elderly living in the community.

## Methods

### Instruments

As proposed by Morgan and Krueger [13], an inductive qualitative research tool was selected. The method of focus group discussion [13] was used with four groups of key-informants: patients, caregivers, health care providers and administrators. Focus groups are an effective method of obtaining data in new or ill-defined research fields. The method is divided in four phases: 1) establishing the questions; 2) planning the focus groups (number and size of groups, time and place of meetings, selection and recruitment of participants, choice of moderator); 3) leading of the focus group; 4) analysis and report. Participants (n = 25) were explained the aim and procedures of the study and agreed by signing a consent form. This research was approved by an ethics committee.

### Recruitment procedure

In order to achieve experimental diversification, participants were identified by purposive selection. The patients (n = 4) and the caregivers (n = 5) were recruited through social workers from the local community service centers, daycare centers and also chosen from a data bank of participants to previous studies. Health care providers (n = 9) were solicited through directors of professional services and coordinators of rehabilitation or home-based services. They worked in different fields of healthcare and services, in rural and urban areas. Administrators (n = 7) were recruited through hospital managers who would identify which one was more familiar with the study clientele and worked in various rural and urban areas. All participants were recruited because of their critical abilities and their experience with needs related to the stroke process from onset to reintegration into the community. The area were the research took place is known as rural and semi-rural with a population of 150,000.

### Data collecting procedure

Each of the four meetings lasted approximately two hours and was recorded on audiotape. A interview guide (Appendix 1 [additional file]) was built according to the method suggested by Morgan and Krueger [13]. Each group included a moderator and her assistant, an observer and a co-investigator. To make sure the participants would convey their perceived and expressed needs, the first meeting was held with the patients' group. Then followed the meetings with the caregivers, the health care providers and finally the administrators. The summary of the first meeting (group of patients) was used as an introduction to discussion for the second group; the third group benefited from the summary of the two previous ones and so forth.

### Data analysis process

In order to ensure the validity of results, data was triangulated as described hereafter. During the last minutes of the meetings, the assistant-moderator would ask the participants to clarify some statements she had jotted down during the encounter. She would then sum up the meeting to validate its content with the participants, at which time they could complement, rectify or add to the information already given. This information along with the notes of the observer would help summarize the focus after each of the meetings. Later, a member of the team listened to the audiotape, allowing further analysis of the data. In the following weeks, each participant was sent a brief report of his/her focus group meeting. One week later, each participant was contacted to corroborate the content of the report. During these interviews, the comments of the participants testified to their understanding and approval of each report and allowed to add some details. Verbal and written summarized reports on the four groups were presented to the research team.

Normative and expressed needs were grouped according to the twelve categories of the HPP model. After analysis of the focus groups, two categories were added in order to include psychological-emotional needs as well as psychological-cognitive needs and the education and work categories were abandoned. The participants from the four groups identified their needs as: a) needs already fulfilled or not mentioned, b) needs partially fulfilled c) unfilled needs.

## Results

Characteristics of the participants of the four focus groups are presented in Table 1. Patients were aged between 71 and 85 years old and most of them had suffered a stroke less than three years before and were identified as having severe to moderate limitations requiring more than 5 hours of assistance per week. None received home-based care from public services and only one in four was assisted by a relative about two hours a week. Most of the caregivers were retired women – aged between 42 and 60 years old – who took care of their disabled husband. Notice that the participants from this group were not related to the participants from the patients' group. Generally, the number of weekly care-giving hours varied from 8 to more than 20 hours.

**Table 1:** Description of characteristics of patients and caregivers

**Patients characteristics**	n = 4	**Caregivers characteristics**	n = 5
**Age**:		**Age**:	
71–75 years old	3	41–59 years	3
81–85 years old	1	60–69 years	2
**Time since stroke**:		**Current occupation**:	
2–3 years	3	Work outside home	1
4–8 years	1	Retired	4
**Homecare public services**		**Relationship with the stroke victim**:	
Yes	0	Wife	4
No	4	Daughter	1
**Help from relatives:**		**Length of caregiver's role:**	
Yes	1 (2 hrs/wk.)	3–4 years	2
No	3	More that 4 years	3
**Level of education**:		**Hours of weekly help**:	
Primary	2	8–12 hours	1
High-school	2	12–16 hours	1
		16–20 hours	1
		More that 20 hours	2
		**Type of help provided**:	
		A.D.L.	5
		I.A.D.L.	5
		Psychological support	3
		Other (stimuli)	3

ADL: activities of daily living

IADL: instrumental activities of daily living

Eight out of nine health care providers had a fairly good knowledge of stroke and seven of them had more than nine years of experience with a stroke clientele. They were from different professional fields: special education (1), occupational therapy (1), social work (2), neuropsychology (1), speech therapy (1), physiotherapy or physical rehabilitation therapy (3). These professionals practiced in an active care hospital (2), a CLSC (4), a daycare center (2), a day hospital (1) and a community organization (1). The administrators came from the same kinds of facilities with the addition of an intensive functional rehabilitation unit and a rehabilitation center. Four of the administrators had a restricted knowledge of stroke and had other customers aside from stroke patients. Two out of seven took care only of persons over 65 years old or with neurological problems. Education fields of the administrators were either management or health related disciplines.

The most important expressed needs of the *patients *were acceptance of their health problem (stroke) and accessibility to physiotherapy and occupational therapy services on an outpatient basis. Then subsequently followed the needs relating to adapted means of transportation, medical follow-up, home visit from healthcare personnel, and stimulus and motivation provided by a caregiver. Domestic help and encouragement from the healthcare personnel were also important needs that have been expressed. Patients were more communicative about their needs relating to community, their psychological and emotional needs, and house alteration requirements. Unfulfilled needs mainly included the occasional home visit from a health professional, domestic help, coaching by the CLSC and medical follow-up.

The foremost rehabilitation need identified by the *caregivers *was that the patient be loved, surrounded, and that he felt secure. These were followed by the necessity to make home adaptations, the need to inform the stroke patient and to provide him with physical and mental stimuli. Finally, the needs relating to tactile sensation problems, supportive care and attention, and acceptance of the situation were also important. It is worth noting that participating caregivers looked after individuals much more severely impaired than our group of patients. These caregivers mentioned the same categories of needs but in a different order of priority. For them, the psychological needs were the top priority; then came the need to adapt the home followed by community living needs. Notice that the beneficiaries had not resumed many of their social roles in the community. According to the caregivers, the less fulfilled needs were mainly related to psychological needs and associated with community living. Specifically, they concerned medication and its side effects, adaptation to this new situation for the caregiver and acceptance by the beneficiary. Respite, emergency help, supportive care and attention, training of family members were also needs partially fulfilled in these categories.

For the *health care providers*, the needs that were less fulfilled mainly concerned related to community living, psychological needs and speech impairment. More precisely, they included: leisure activities, awareness, long-term family support, bond between spouses, respect of the person's pace, delivery of timely and simplified medical information to the patient and his/her spouse, adaptation of the home and respite, because in rural areas, for instance, there is no specialized transportation.

Re-education for basic activities like eating or getting dressed and psychological assistance for acceptance, self-esteem and dignity were the most important. Followed the capacity to communicate and the family's need to be supported in understanding the health problem. Finally, long-term follow-up, simplification of the information given and home alterations also constituted essential needs, again according to the health care providers. For the most part, the health care providers worked with severely disabled persons. In their view, the interventions having to do with personal care held a priority over those concerning psychological needs and communication needs. As also identified by the caregivers, needs related to community living and the home followed.

In the *administrators*' view, a psychological support intervention made by the case manager, for instance, was more necessary in rural areas. They mentioned more "partially fulfilled" needs in the areas of speech, mobility and community living. They underlined the necessity to become sensitive to this clientele with cognitive problems and their needs for spirituality, means of transportation, financial support, the need to maintain and improve speech re-education.

Administrators emphasized their wish to ensure the complete management of the patient. They were interested in the list of needs identified by the patients, since the needs of the affected individuals cannot be dissociated from those of their family. Needs identified as a priority by the administrators were a stabilized health condition, recovery of biological, psychological and social abilities, compensation mechanisms, integration into the community and support in finding a new sense to one's life. The chronology of those needs was unanimous because it set a continuum in the rehabilitation process. Priority needs identified by the administrators seemed closer to those listed by the patients. Administrators seemed to believe the patients had made a fairly good functional recovery and insisted on needs related to community living, personal care and psychological well-being. Finally, they were the only ones to clearly talk about spiritual needs.

On the whole, unfulfilled needs were identified in the four groups as from categories relating to housing, community living, psychological and emotional needs. Table 2 summarizes the response to rehabilitation needs according to the categories of from the HPP model.

**Table 2:** Participants' perception of the response to rehabilitation needs

	**Patients**			**Caregivers**			**Health care providers**			**Administrators**		
	
	**F**	**PF**	**U**	**F**	**PF**	**U**	**F**	**PF**	**U**	**F**	**PF**	**U**
**Nutrition**		x		nm			nm			nm		
**Body condition**		x				x		x			x	
**Personal care**		x		nm				x			x	
**Communication**	nm					x		x			x	
**Housing**		x			x			x		x		
**Mobility**		x		nm				x			x	
**Responsibilities**	nm			nm			nm			nm		
**Interpersonal relationships including sexuality**			x		x			x			x	
**Community living**			x		x				x		x	
**Leisure activities**	nm				x			x		x		
**Psychological**			x			x		x		x		
**Cognitive**			x			x		x		nm		

**F**: Need fulfilled; **PF**: Need partially fulfilled; **U**: Need unfulfilled; nm: Need not mentioned by this group of participants Note: the «education» and «work» categories were removed.

Although some of the categories were partially fulfilled for the health care providers and administrators, the gradient of the providers' opinion on each of the needs specifically mentioned, stood between "partially fulfilled" and "unfulfilled". Conversely, the gradient of the administrators' answers stood between "fulfilled" and "partially fulfilled". Interestingly, only the patients' group acknowledged nutrition needs.

## Discussion

In Lewinter et al. [14], results from individual interviews show that the cognitive needs are not very well fulfilled, if at all, by the available rehabilitation services. Results from the current study tend to draw the same conclusions. Indeed, the patients from the groups mentioned they had to perform their own intellectual activities, in order to stimulate and maintain cognitive functions during rehabilitation and after reintegrating their home. Furthermore, the patients from the current study point out the lack of continuity between resources when discharged from rehabilitation services. This also corroborates the results of Zwygart-et al [15], who collected their data through a mail questionnaire, and those of Brandriet, et al. [16].

The perceived needs of the caregivers really stood out during focus groups and would be inseparable from those of the patients. Caregivers considered rehabilitation in terms of recovery and excluded compensation mechanisms. For instance, they were unable to imagine any possible means to compensate for poor vision, a need expressed by the patients. While patients referred to the psychological need of accepting the situation, caregivers mentioned the psychological need to face one's own limitations and the probability of complete rehabilitation. These results agreed with the study of Gauthier [17] who collected his data six to nine months post-stroke, and who noticed some unfulfilled psychological needs.

From our results, it seems that supporting the caregiver at the time of the hospitalization would allow him to offer a better support to the stroke victim. That support would perhaps allow acceptance of the situation and an active implication in the rehabilitation. Long-term follow-up and respect of the person's pace were mentioned as being important. Caregivers felt useful and appreciated this phase of the rehabilitation where attendance, supportive care and attention as well as information were provided at the day hospital. All groups pointed out the need for a more aggressive early re-education and a continuing rehabilitation. None of the groups mentioned needs pertaining to responsibilities. Finally, needs associated with sexuality were acknowledged by all groups, but none offered any solution.

Health care providers felt powerless, even frustrated, with so many needs to be fulfilled and so little human resources available. Health care providers proposed many possible avenues worth exploring in relation with community and some ways of addressing psychological problems.

The health care providers identified most of the partially fulfilled needs, ranging the whole continuum of the rehabilitation process. They pointed out the necessity for health system administrators to acknowledge expressed and normative needs of stroke patients. Adding human resources would begin solving the problem. Patients who benefit from intensive rehabilitation (about 10% of the stroke clientele) are treated according to their impairments in order to diminish their disabilities and improve their functionality. Services to help the family and to modify to the physical environment are usually available. The only help rarely provided is that of fulfilling psychological or cognitive needs and supporting social integration. However for 90% of the stroke clientele who only benefits from rehabilitation services at the acute care hospital, little of their needs are fulfilled.

This data concurs with that of Lewinter et al. [14] on the inadequate length of rehabilitation services, as reported by patients and caregivers. As inferred by these focus group discussion, it would appear more important, in the current health system, to assess capabilities and impairments of the stroke patients than to offer proper services to fulfill their expressed needs. And yet, the same health care providers considered the assessment more important to their own normative needs than to those of the patients.

In general, the answers of the administrators were based on what the resources should offer instead of on the accessibility to services, which they were less aware of. It seems that most of the participating administrators were preoccupied by the needs of the patients throughout the whole process and not only when they were using their services. They proposed solutions focused on the needs of the patient and on the most adequate resource to fulfill those needs at this stage of the rehabilitation process. In fact, the need for specially trained personnel to help prevent contractures and dehydration, as well as spiritual needs, were reported by administrators from chronic care facilities. Spiritual needs had also been reported by McLean et al. [18] in their pilot study with individual interviews. Administrators were much more precise in their description of the patients' sexual needs than the health care providers. This need was also described in the Lewinter et al. [14] study, but solutions have yet to be proposed. Since they are often consulted on this topic, the administrators were surprised the health care providers had so little to say about it. Admitting that the providers may be uncomfortable about their patient's sexuality, administrators sometimes provide counselling themselves.

Regarding prevention and treatment of secondary impairments, which is the second stage of the rehabilitation process described by Duncan, et al. [19], administrators pointed out the importance of intense early rehabilitative interventions, no matter how old the stroke victim was. They noticed gaps in the early rehabilitation process that bear long-term repercussions. They identified a lack or resources or of specific knowledge as possible reasons of these impairments in those services where the priority is to have versatile care providers to work with a diversified clientele. They also mentioned the importance of integration to promote long-term rehabilitation. They seemed to trust existing services and the competence of health care providers regarding functional rehabilitation and compensation needs. Even though the administrators said that all services were available, they acknowledged the necessity of a resource person to help access these services. In a system where, it seems, the responsibility of answering the needs of stroke patients is being discarded, case managers and administrators will share this difficult task of making services available and guiding the patients towards them. It is interesting to note the discrepancy between the perception of fulfilled needs identified by the patients and the caregivers and those identified by the health care providers and administrators. This inconsistency shows that the two categories of needs in Bradshaw's taxonomy [9], namely the perceived and expressed needs (patients and caregivers) are different from the normative needs (health care providers and administrators).

### Strengths and limits

Many strengths and limitations have to be mentioned. Methodologically speaking, holding the first meeting with the patients' focus group ensured a valid identification of their needs, as they were perceived. As for the other participants, recruitment was done in a rigorous manner in order to obtain a fairly equal number of representatives from rural and urban areas, from various professions, and from different types of rehabilitation resources, including representatives of community organizations. It should be mentioned that recruitment of the patients and caregivers was difficult; participants from these two groups were probably not typical of persons being dismissed to their home after a stroke. However, the fact that the severity of the stroke ranged from severe to mild in those two groups increases the external validity of the results.

Recall bias is significant in this study as patients and caregivers had to recall events that took place few years ago.

The implication of the four groups of participants ensures a better validity of the data as was suggested by Liu and Mackenzie [20]. The rigorous triangulation analysis also ensures a good validity of the results. Data collection having been done more than two years after the stroke allowed for identification of needs in the continuing process of rehabilitation, until social integration. The obvious needs concern the actual day-to-day living of the elderly, because the study was intended for stroke patients who were over 65 years old when they had their stroke. During the focus group discussions, patients who had suffered a stroke at least two years before conveyed their perception of unfulfilled needs all through the rehabilitation process. However, the "unfulfilled" perception may have been attributable to memory loss. Nevertheless, since the results from this group of participants agree with those from the caregivers' and health care providers' groups, and sometimes even with those of the administrators' group, it seems that the cause is to be attributed to the inaccessibility to services, their unavailability or a poor knowledge of their existence. To help clarify this question, a multi-center longitudinal study is ongoing to follow-up on the evolution of the needs in rehabilitation for post-stroke victims after being released to their home.

## Conclusions

After a stay in an active care hospital (ACH), before the patient is discharged, a meeting between family members and a stroke specialist (social worker, nurse and volunteer worker having had similar experience) could allow discussions about a possible mental depression of the stroke patient and about upcoming difficulties. It would also be necessary at this time to give the caregivers information on their loved one's health status and to inform them of their future needs.

After hospital discharge, a specially trained healthcare worker could remain available by telephone to counsel the caregivers. Finally, a psychological follow-up of the caregiver would allow him to better support his/her relative in his/her rehabilitation process. After discharge from the hospital or from the rehabilitation center, a better communication between healthcare facilities and increased availability of services at the CLSC – like twice a year home visits by a health care provider – are suggested by the patients. As concluded by Lewinter et al [14], it seems that a professional psychological follow-up to discuss acceptance and consequences of non-acceptance would favour mid- to long-term rehabilitation.

### Recommendations to health services

Improving accessibility to services, respecting priority parking spaces for the disabled and promoting public cooperation would allow for a better social integration and recovery of social roles. On the whole, a better distribution of financial resources between institutions having to deal with this clientele, would allow long-term support of the person and his/her family. Health care providers first suggest that full rehabilitation teams (from different professional fields) be created (for example stroke team). They also propose the appointment of a case administrator or pivotal health care provider, based on the total system concept in healthcare, which would simplify communication between partners (hospitals, local community service centers, rehabilitation centers, daycare centers...). Services would be more suited to the needs of the individual and available for the families. Intervention priorities would be centered on needs expressed by the person. Education programs on how to deal with different types of clienteles would be made available to the health care providers to help them adjust to the difficulties associated with all kinds of issues. For mid- to long-term needs, improving means of transportation, adding support groups in rural areas, improving long-term follow-up in urban areas, humanizing of care, making information accessible, educating the neighbours and demystifying stroke by informing the public, would help integration and recovery of social roles to counter handicap situations. Finally, it is imperative to consider the perceived and expressed needs of the patients and caregivers and to integrate those needs with the normative needs identified in planning rehabilitation programs.

## Supplementary Material

Additional File 1 Appendix 1: Course of the group discussions for the patients, caregivers, health providers and administrators Click here for file
