# Supplementary material for: Identification of rehabilitation needs after a stroke: an exploratory study
Source: Health Qual Life Outcomes. 2004 Sep 21;2:53. doi: 10.1186/1477-7525-2-53 (PMC524186; doi:10.1186/1477-7525-2-53)
Supplement: Additional File 1 — Appendix 1: Course of the group discussions for the patients, caregivers, health providers and administrators [file 1477-7525-2-53-S1.doc]

# Additional File - Appendix 1: Course of the group discussions for the patients, caregivers,

# health providers and administrators

Demographics / Consent forms / Travel expenses

| 10: 00 |  | Word of welcome by monitors and observers:   - Object of meeting   Identification of rehabilitation needs of stroke sufferers related to their discharge to their home from the hospital up until now (following a pilot study)   - Course: Tight schedule. Apologize if people feel rushed. - Participation of everybody. Talk loud, one at a time and avoid putting things on the table. |
| --- | --- | --- |
| 10: 05 | Q1 | Can you introduce yourself and tell us what is your favourite hobby (or activity that you pass time with) |
| 10: 10 | Q2 | What does rehabilitation mean for you? |
| 10: 15 | Q3 | What if I tell you that needs are the difference between what you wish and what is your current situation (for example, being confined in your home). Here is a list of rehabilitation needs identified by patients after a stroke, from hospital to their return home. We will go through them together and we are asking you to classify them as either fulfilled (F), partially fulfilled (PF) or unfulfilled (U) needs. (Give examples if needed to validate your choices). |
| 10: 55 | Q4 | Are there rehabilitation needs you think of that did not come up in prior group discussions?  (Be careful to separate needs and rehabilitation services. When a service is mentioned, make sure you know to what need it would apply) |
| 11: 05  Break |  | We will take a break and during the break, we are asking you to look at the Flip Charts sheets and tell us if these needs should be added to your list. |
| 11: 15 | Q5 | Lets examine one-by-one, the needs that are partially fulfilled and unfulfilled; can you find a solution to allow them to be fulfilled? |
| 11: 35 | Q6 | I would ask you to choose three needs that seem the most important to you while in hospital, rehabilitation, back home |
| 11: 40 |  | Summary by the assistant-moderator |
|  | Q7. | Does this summary accurately reflect what was said or discussed? |
| 11: 45 | Q8. | The objective of this discussion was to identify the rehabilitation needs of stroke sufferers, in relation with their continuing living at home.  Following these discussions, would you like to add anything that you forgot and consider important? |
| 11: 50 |  | Acknowledgements |
| 11: 55 |  | End of meeting / Departure |
